# Neutrophil *Myo5c* gene downregulation is associated with postoperative organ dysfunction following pediatric cardiac surgery with cardiopulmonary bypass

**DOI:** 10.3389/fcvm.2025.1380606

**Published:** 2025-05-27

**Authors:** Wiriya Maisat, Sumiti Sandhu, Samuel Kim, Hanna Van Pelt, Sek Won Kong, Juan Ibla, Koichi Yuki

**Affiliations:** ^1^ Department of Anesthesiology, Critical Care and Pain Medicine, Boston Children’s Hospital, Boston, MA, United States; ^2^ Department of Anaesthesia, Harvard Medical School, Boston, MA, United States; ^3^ Department of Immunology, Harvard Medical School, Boston, MA, United States; ^4^ Department of Anesthesiology, Faculty of Medicine Siriraj Hospital, Mahidol University, Bangkok, Thailand; ^5^ Computational Health Informatics Program, Boston Children’s Hospital, Boston, MA, United States; ^6^ Department of Pediatrics, Harvard Medical School, Boston, MA, United States; ^7^ Broad Institute of Harvard and MIT, Cambridge, MA, United States

**Keywords:** congenital heart disease, cardiopulmonary bypass, organ injury, *myo5c*, neutrophil extracellular traps

## Abstract

**Introduction:**

Pediatric cardiac surgery with cardiopulmonary bypass (CPB) carries substantial risks of postoperative organ dysfunction and mortality, making the identification of biomarkers for postoperative organ dysfunction crucial for enhancing patient outcomes. As neutrophils play a major role in the perioperative setting and act as double-edge swords to the host, we examined neutrophil transcriptomic profiles in pediatric patients undergoing cardiac surgery with CPB.

**Methods:**

We enrolled into this study from May 31, 2022, to February 22, 2023.

**Results:**

32% developed postoperative complications, mainly in the respiratory and cardiovascular systems. Patients in the complication group showed higher PELOD-2 scores on postoperative day 2. These patients experienced longer duration of mechanical ventilation and extended ICU and hospital stays. RNA sequencing of neutrophils revealed significant changes in gene expression after CPB, with the *myo5c* gene emerging as a key downregulated transcript. Its expression was inversely correlated with PELOD-2 score, IL-6 levels, and markers of neutrophil and platelet activation. Furthermore, *myo5c*-knockout HL60 cells exhibited enhanced neutrophil extracellular traps (NETs) formation upon stimulation, supporting a potential regulatory role for *myo5c* in neutrophil activation and systemic inflammation.

**Discussion:**

While *myo5c* was not an independent predictor of complications, its expression was consistently associated with clinical severity, suggesting it may serve as a useful biomarker for early risk stratification of postoperative complications in this vulnerable pediatric population.

## Introduction

Cardiac surgery for patients with congenital heart diseases (CHDs) is notably at high-risk of organ injury and mortality. Particularly neonates and infants undergoing cardiac surgery remain associated with the highest morbidity and mortality due to organ injury/failure (in-hospital mortality of 6.9%) (1, 2). In our previous investigation, neonates/infants had the highest rate (17.1%) of thrombosis and multiple organ dysfunction with the mortality rate of 4.3% (3). Identifying predictive markers for postoperative outcomes and developing intervention strategies is crucial for improving patient care.

Neutrophils, as first-line immune responders, are responsible for both microbial clearance and facilitating tissue repair (4). Thus, they are highly activated in the perioperative setting. While this activation is essential for immune defense, it can also paradoxically contribute to organ injury (5). Neutrophil extracellular traps (NETs), one of the strategies for neutrophils to eradicate pathogens (6), can contribute to microvascular thrombosis and organ injury (7, 8), complicating the postoperative recovery process.

This study focused on neutrophil transcriptomic profiles in pediatric patients undergoing cardiac surgery with cardiopulmonary bypass (CPB). The aim was to determine specific genetic markers that could serve as indicators of postoperative organ injury. The ultimate goal was to enhance postoperative management in pediatric cardiac surgery, mitigate the risk of organ injury, and improve overall patient outcomes.

## Method

### Study design

This prospective cohort study was conducted at a quaternary academic pediatric medical center. The study was approved by the Institutional Review Board of Boston Children's Hospital (Protocol number IRB-P00033314, approval date 02/18/2020). Written informed consent for participation was obtained from a parent or legal guardian.

### Patient selection and perioperative course

All patients scheduled for CHD with CPB at our center were considered for inclusion. We excluded patients who had active infections, were on chronic steroid therapy, had immunodeficiencies, HIV, or a history of malignancy. The study period spanned from May 31, 2022, to February 22, 2023. Included patients received general anesthesia with endotracheal intubation and had arterial and central venous lines placed. Following surgical dissection, they were heparinized and cannulated for CPB. The CPB circuit was primed with red blood cells (RBC) and fresh frozen plasma (FFP) to maintain a hematocrit level above 30%, following our institutional protocol. RBC priming was selectively applied to patients with anticipated hemodilution risk, particularly those under 10 kg. All transfused RBC units were pre-storage leukocyte-depleted and used within 7 days of collection to minimize storage-related inflammatory activation. Circulatory arrest, regional perfusion, temperature management, and modified ultrafiltration (MUF) were used as needed. Platelet and cryoprecipitate were administered for non-surgical microvascular bleeding. Typically, neonates and infants did not receive FFP post-CPB in our institution. At the conclusion of surgery, patients were either extubated or remained intubated based on the anesthesiologist's decision and transferred to the ICU for further care.

### Data collection

For the analysis of clinical data, we extracted a comprehensive range of data from electronic medical records. This included demographic details, comorbidities, diagnoses, surgical procedures, laboratory results, specifics of CPB, and details of postoperative complications. Additionally, data on mechanical ventilatory support, vital signs, and the duration of ICU and hospital stays were collected. It has been increasingly recognized that morbidity and mortality in the ICU are due to multiple organ failure (9). Frequently used methods to assess pediatric severity of illness in the pediatric ICUs include the Pediatric Logistic Organ Dysfunction score (PELOD), the Pediatric Risk of Mortality score (PRISM III), and the Pediatric Index of Mortality score (PIM2) (10–13). The comparison of the three scoring systems for mortality prediction demonstrated that the receiver operating characteristic curve (ROC) was 0.98 for PELOD (12), 0.900 for PIM-2 (11) and 0.82 for PRISM3 (9). In addition, PELOD has been used widely in pediatric CHD population (14). Accordingly, we used PELOD in this study to score organ function. PELOD-2 score was manually calculated as previously described (15). We also utilized the Society of Thoracic Surgeons—European Association for Cardio-Thoracic Surgery Congenital Heart Surgery Mortality Categories (STAT Mortality Categories) to examine the risk of morbidity associated with surgical procedures. Postoperative complication was defined as organ dysfunction/injury or thrombosis. We adopted the criteria for organ dysfunction as previously described in pediatric critical illness (16) along with PELOD-2 as described above. Thrombosis was defined as the presence of any vascular thrombosis detected using ultrasound diagnostic imaging.

### Blood sample collection

Blood samples were drawn from patients via existing central venous catheters at two distinct time points: (1) immediately after the induction of anesthesia, serving as the baseline, and (2) upon admission to the ICU. The volume of blood collected at each time point adhered to the guidelines specified in the “Protection of Human Subjects” document, totaling 1.8 ml for both collections (900 µl each). After sample collection, blood was immediately transported to the laboratory for assays. For each time point, 100 µl was used for flow cytometry analysis, and 800 µl was subjected to plasma and neutrophil purification. Flow cytometry analysis and RNA sequencing experiment methods are included in Supplementary document.

### Inflammatory cytokine measurement

Plasma cytokine levels, including TNF-α, IL-1β, IL-2, IL-4, IL-6, IL-8, IL-10, IL-12p70, and IL-13, were measured using V-plex proinflammatory panel 1 human kit (Meso Scale Discovery; Gaithersburg, Maryland), in accordance with the manufacturer's protocol.

### Statistical analysis

For the analysis of clinical data, we utilized IBM SPSS Statistics, Version 30.0 (IBM Corp., NY, USA). Categorical variables were presented as numbers and percentages, while continuous variables were summarized as means and standard deviations for normally distributed variables, or medians and interquartile ranges for skewed distributions. The normality of variables was assessed using the Shapiro–Wilk test. Categorical variables were compared using chi-square test, Fisher exact test, or Kruskal–Wallis test as appropriate. Continuous variables were analyzed using Student's *t* test or Mann–Whitney *U* test, depending on their distribution. Logistic regression models were constructed to assess whether the association between *myo5c* expression (at baseline or ICU admission) and postoperative complications was confounded by clinical variables. Variables with a *p*-value <0.05 in univariable analysis were included in the models as potential confounders. For correlation analysis, Pearson or Spearman correlation coefficient was determined based on the normality of the data. Laboratory data were analyzed as outlined in the corresponding figure legends. Statistical significance was set at *p* value < 0.05. All statistical analyses were performed using Prism 10 software (GraphPad Software, La Jolla, CA, USA).

## Results

### Pediatric patients undergoing cardiac surgery demonstrated a high incidence of postoperative complications

We enrolled 50 patients undergoing congenital cardiac surgery with CPB between May 31, 2022–February 22, 2023. Table 1 presents the demographic data and baseline characteristics of the patient cohort. Median age was 5.8 months with 42% being male.

**Table 1:** Demographic and clinical characteristics of all patients.

Covariates	All patients
	(*n* = 50)
Gender: male	21 (42.0)
Age (mo)	5.8 (1.3,24.2)
Body weight (kg)	6.9 (3.9,12.1)
ASA classification	
3	10 (20.0)
4	40 (80.0)
Diagnosis	
Septal defect	4 (8.0)
IAA/aortic arch hypoplasia	8 (16.)
TOF	2 (4.0)
PA/PS	2 (4.0)
Aortic valvular disease	4 (8.0)
Complete AVSD	3 (6.0)
DORV	3 (6.0)
HLHS	5 (10.0)
Single ventricle, other	3 (6.0)
Mitral valvular disease	2 (4.0)
PVS	3 (6.0)
TGA	3 (6.0)
Conduit failure	2 (4.0)
Coarctation of aorta	1 (2.0)
CcTGA	2 (4.0)
Other	3 (6.0)
Associated disease	
Down syndrome	3 (6.0)
Cerebrovascular disease	4 (8.0)
Tracheo/broncho/laryngomalacia	3 (6.0)
Lung hypoplasia	2 (4.0)
DiGeorge syndrome	1 (2.0)
Heterotaxy syndrome	2 (4.0)
Other	2 (4.0)
STAT mortality category	
1	13 (26.0)
2	18 (36.0)
3	8 (16.0)
4	8 (16.0)
5	3 (6.0)

In the table, data is presented as median (interquartile range), or number (percentage). The abbreviations in the Table are as follow.

AVSD, atrioventricular septal defect; ccTGA, congenitally corrected transposition of the great arteries; CPB, cardiopulmonary bypass; DORV, double outlet right ventricle; HLHS, hypoplastic left heart syndrome; IAA, interrupted aortic arch; MUF, modified ultrafiltration; PA, pulmonary atresia; PS, pulmonary stenosis; PVS, pulmonary venous stenosis; RBC, red blood cells; STAT, society of thoracic surgeons-European association for cardio-thoracic surgery congenital heart surgery mortality categories; TGA, transposition of the great arteries; TOF, tetralogy of fallot.

Among these patients, 16 individuals (32%) developed postoperative complications (Table 2). These complications were predominantly respiratory (*n* = 10, 20%) and cardiovascular in nature (*n* = 9, 18%). Notably, eight patients (16%) exhibited multi-system involvement, indicating the complexity of these complications. A significantly higher proportion of patients with complications were in STAT mortality category ≥3 (62.5% vs. 26.5%, *p* = 0.027). RBC transfusion during CPB were administered to 37.5% of patients in the complication group (6 of 16) and 29.4% in the non-complication group (10 of 34) (*p* = 0.57). Postoperative lactate levels tended to be higher in patients with complications (4.1 vs. 2.8), with the difference approaching statistical significance (*p* = 0.055) (Table 2). Additionally, patients with complications had remarkably longer periods of mechanical ventilation (71.0 h vs. 28.0 h, *p* = 0.013), extended ICU stays (166.0 h vs. 72.0 h, *p* < 0.0001), and prolonged hospital stays (14.9 days vs. 8.9 days, *p* = 0.01). These patients exhibited higher PEdiatric Logistic Organ Dysfunction-2 (PELOD-2) scores on postoperative day 2 (4.5 vs. 2.0, *p* < 0.001), indicating a greater overall severity of organ dysfunction (Table 3).

**Table 2:** The comparison of demographic and perioperative characteristics between patients with and without complications.

Covariates	All patients	Complication	No complication	*P* value
	(*n* = 50)	(*n* = 16)	(*n* = 34)	
Age (mo)	5.8 (1.3, 24.2)	4.0 (1.4, 16.4)	5.8 (0.8, 28.4)	0.64
STAT mortality category ≥3	19 (38.0)	10 (62.5)	9 (26.5)	0.027*
ASA classification				
3	10 (20.0)	2 (12.5)	8 (23.5)	0.47
4	40 (80.0)	14 (87.5)	26 (76.5)	
Preoperative mechanical ventilation	13 (26.0)	3 (18.8)	10 (29.4)	0.51
Operative time (min)	356.0 (309.5, 461.0)	386.0 (320.5, 531.0)	353.0 (311.3, 453.8)	0.16
CPB time (min)	170.0 (134.0, 252.5)	235.0 (143.0, 292.0)	170.5 (143.5, 232.3)	0.18
Aortic cross-clamp time (min)	117.0 (82.5, 161.5)	119.0 (72.5, 185.5)	116.5 (85.5, 156.3)	0.84
Circulatory arrest use	11 (22.0)	6 (37.5)	5 (14.7)	0.052
Regional perfusion	12 (24.0)	4 (25.0)	8 (23.5)	0.33
MUF	36 (72.0)	24 (70.6)	12 (75.0)	0.29
RBC transfusion during CPB (no. of patient)	16 (32.0)	6 (37.5)	10 (29.4)	0.57
RBC transfusion during CPB (ml/kg)	0 (0, 8.2)	0 (0, 21.8)	0 (0, 8.2)	0.62
Postoperative RBC transfusion (no. of patient)	18 (36.0)	7 (43.8)	11 (32.4)	0.43
Postoperative RBC transfusion (ml/kg)	0 (0, 120)	0 (0, 15.4)	0 (0, 14.3)	0.55
Postoperative lactate	2.4 (1.8, 4.1)	4.1 (3.0, 6.3)	2.8 (2.3, 3.6)	0.055

In the table, data is presented as median (interquartile range) for continuous variable, or number (percentage) for categorical variable. For continuous variable, statistical analysis was performed using Mann–Whitney *U* test. For categorical variable, chi-square test was used.

The abbreviations in the table are as follow.

CPB, cardiopulmonary bypass; MUF, modified ultrafiltration; RBC, red blood cells; STAT, society of thoracic surgeons-European association for cardio-thoracic surgery congenital heart surgery mortality categories.

* *P* < 0.05 was considered significant.

**Table 3:** The profile of postoperative outcomes after pediatric cardiac surgery.

Covariates	All patients	Complication	No complication	*P* value
	(*n* = 50)	(*n* = 16)	(*n* = 34)	
Duration of mechanical ventilation (h)	44.4 (20.7, 74.8)	71.0 (27.5, 187.3)	28.0 (20.4, 68.7)	0.013
ICU stay (h)	93.5 (48.0, 162.3)	166.0 (92.0, 323.0)	72.0 (44.8, 127.5)	<0.0001*
Hospital stay (d)	9.9 (8.0, 19.0)	14.9 (11.9, 32.8)	8.9 (6.0, 12.0)	0.001*
Number of complication				
≥2	8 (16.0)	-	-	-
1	8 (16.0)			
0	34 (68.0)			
Type of complication				
Respiratory dysfunction	10 (20.0)	-	-	-
Cardiovascular dysfunction	9 (18.0)			
Neurologic dysfunction	3 (6.0)			
Coagulopathy	3 (6.0)			
Renal dysfunction	0 (0)			
Hepatic dysfunction	0 (0)			
Thrombosis	2 (4.0)			
PELOD-2 score				
Postoperative day 1	5 (5, 5)	3.5 (3.0, 5.0)	5.0 (5.0, 5.0)	0.001*
Postoperative day 2	2 (2, 3)	4.5 (3.0, 5.8)	2.0 (2.0, 2.0)	<0001*
In-hospital death	1 (2.0)	1 (6.3)	0 (0)	-

In the table, data is presented as median (interquartile range) for continuous variable, or number (percentage) for categorical variable. For continuous variable, statistical analysis was performed using Mann–Whitney *U* test. For categorical variable, chi-square test was used.

The abbreviations used in the table are as follow.

ICU, intensive care unit; PELOD-2, pediatric logistic organ dysfunction-2.

* *P* < 0.05 was considered significant.

### RNA sequencing analysis demonstrated inflammatory responses and cellular stress in neutrophils in the early postoperative period following congenital cardiac surgery with CPB

Neutrophils are the first responders to inflammation/ stress during the perioperative phase, exhibiting an increase in numbers and activation in response to surgical triggers and associated inflammatory stimuli. Given this physiological response, we posited that variations in neutrophil RNA transcription might distinguish patients with postoperative complications from those without. To test this hypothesis, we performed RNA sequencing (RNA-seq) analysis of neutrophils, both at baseline and upon admission to the ICU.

**Figure 1:**
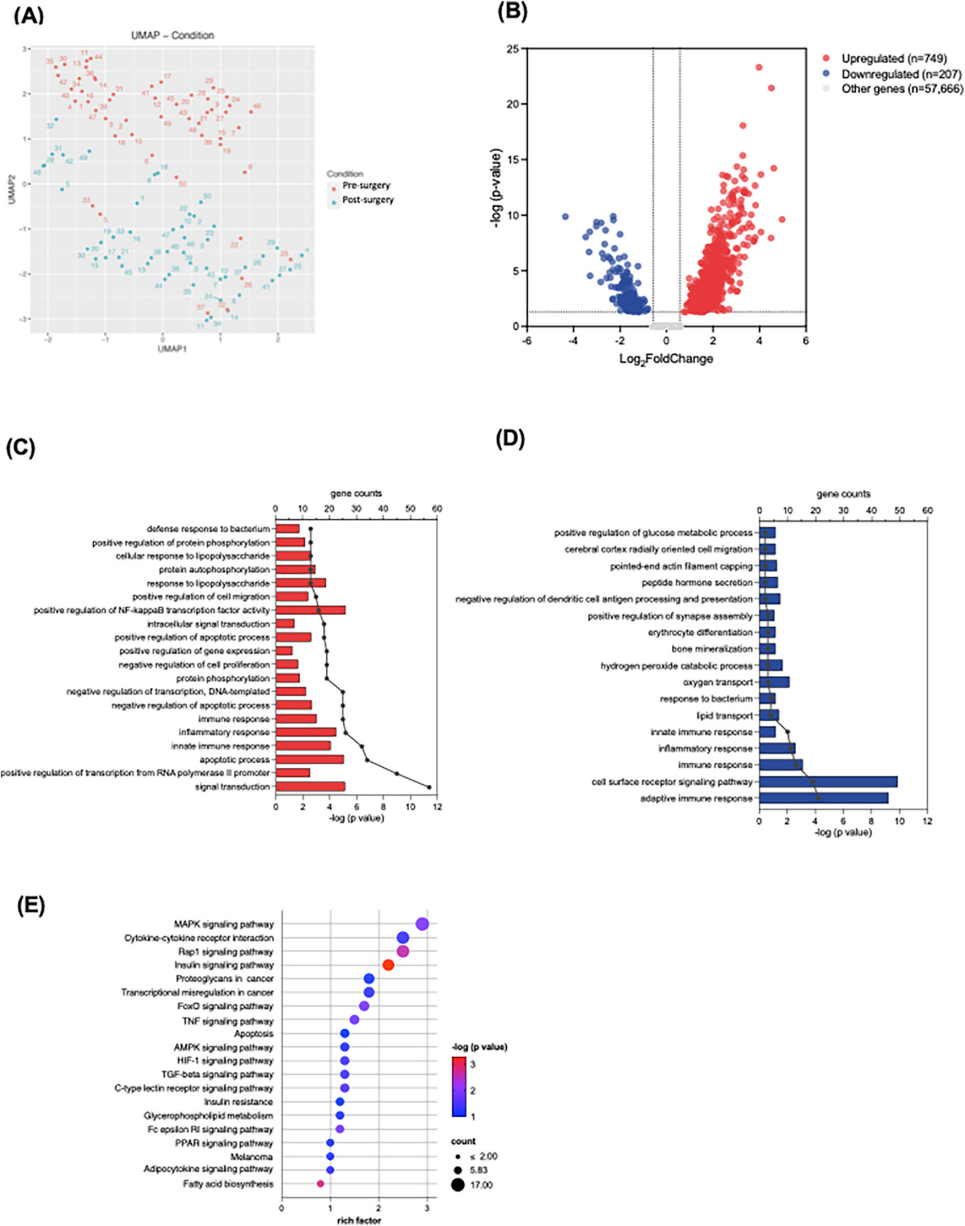
RNA sequencing analysis of neutrophils at baseline and upon ICU admission. **(A)** UMAP plot demonstrates clusters between the baseline (blue) and ICU admission (red) time points. **(B)** The volcano plot shows upregulated and downregulated genes; the dotted line represents the threshold of differential gene screening criteria. **(C,D)** Gene ontology (GO) of biological process enrichment analysis of top 20 significantly upregulated **(C)** and downregulated **(D)** differentially expressed genes (DEGs). Bar plots represent –log (adjusted *p* value) calculated using the Benjamini-Hochberg correction. Line and dotted plots represent gene counts for the corresponding GO terms. **(E)** KEGG pathway enrichment analysis of upregulated DEGs. Rich factor refers to the ratio of the number of DEGs to the number of total annotated genes in the pathway. The point color represents –log (adjusted *p* value) using the Benjamini-Hochberg correction, and the point size represents the number of DEG mapped to the reference pathway.

In our RNA-seq analysis, we utilized the Uniform Manifold Approximation and Projection (UMAP) technique to visualize the complex gene expression data in high dimensions. The resulting UMAP plot (Figure 1A) distinctly delineated clusters between the baseline and ICU admission time points, indicating significant alterations in RNA expression patterns attributed to the cardiac surgery and CPB procedure.

The bulk RNA-seq analysis identified a total of 58,622 transcripts. We used criteria of an absolute log_2_ fold change >0.58 and a statistically significant adjusted *p*-value < 0.05 to determine DEGs at ICU admission compared to baseline. The volcano plot illustrated the 749 upregulated and 207 downregulated DEGs (Figure 1B). Further exploration on these DEGs included GO enrichment analysis, primarily focusing on GO Biological Processes (GO-BP). Within the top 20 enriched GO-BP terms associated with upregulated DEGs, we observed notable enrichment in various inflammatory processes, such as apoptotic processes, innate immune responses, and the regulation of nuclear factor-kappa B (NFκB) transcription factor activity (Figure 1C). Conversely, the adaptive immune response appeared to be downregulated in the early postoperative period (Figure 1D). Our KEGG pathway analysis further demonstrated the upregulated DEGs involved several inflammatory signaling pathways and apoptosis, encompassing pathways such as mitogen-activated protein kinase (MAPK), Ras-associated protein-1 (Rap 1), hypoxia-inducible factor (HIF)-1 and tumor necrosis factor (TNF) signaling pathways (Figure 1F). These findings substantiate the presence of robust inflammatory responses in the neutrophils during the early postoperative period following congenital cardiac surgery with CPB.

### Neutrophil myo5c gene was downregulated in patients exhibiting high PELOD-2 scores on postoperative day 2

**Figure 2:**
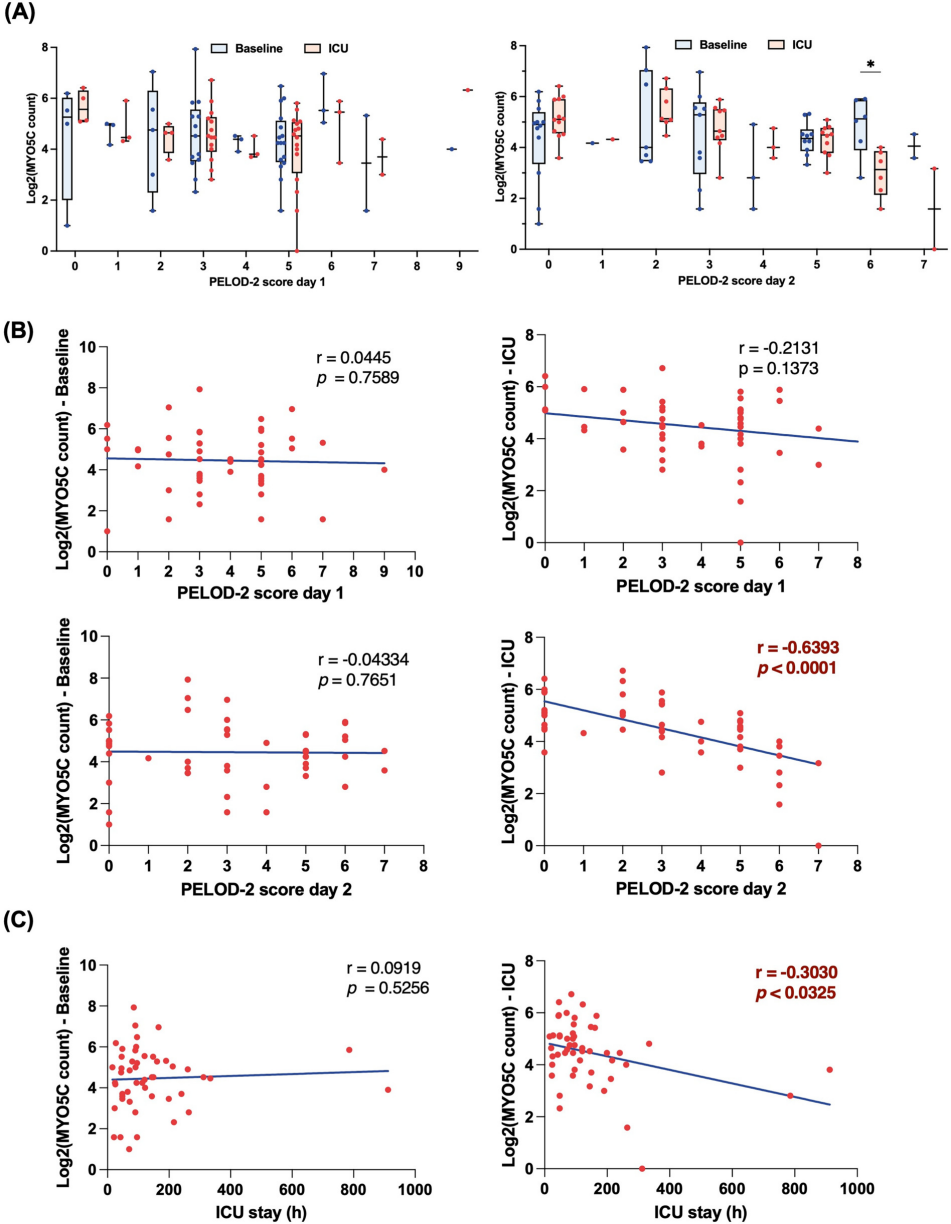
Downregulation of *myo5c* gene expression in neutrophil is associated with high PELOD-2 score on postoperative day 2. **(A)** Comparison of *myo5c* gene expression in neutrophil between baseline (blue) and upon ICU admission (red) at different PELOD-2 score on postoperative day 1 (left panel) and day 2 (right panel). Each dot indicates raw data. Data are presented as median (IQR). Statistical analysis was performed using mixed-effect model. The Bonferroni correction was applied to adjust *p*-values for multiple comparisons. **(B)** Correlation analyses between *myo5c* gene expression in neutrophil and PELOD-2 score at different time point. Statistical analysis was performed using Spearman correlation analysis between *myo5c* expression levels and PELOD-2 score. Spearman correlation analysis was used, and correlation coefficients (*r*) and corresponding *p*-values are shown. **(C)** Correlation analyses between *myo5c* gene expression in neutrophil and ICU stay (h) Statistical analysis was performed using Spearman correlation analysis between *myo5c* expression levels and ICU stay (h) Spearman correlation analysis was used, and correlation coefficients (*r*) and corresponding *p*-values are shown.

To delineate the association between neutrophil mRNA expression and postoperative complications, we employed the PELOD-2 score as a surrogate marker for postoperative complications, capitalizing on its well-established correlation with pediatric organ dysfunction and its ability to provide a broader range for a more nuanced understanding of complications (15). Among several candidate genes identified from the RNA sequencing dataset, *myo5c* was the only transcript that consistently correlated with indicators of postoperative organ dysfunction, including PELOD-2 scores. Although the scores were assessed for both postoperative days 1 and 2, the association with *myo5c* expression was more pronounced and statistically significant on day 2, which was therefore prioritized in our analysis. Our analysis showed a notable reduction in *myo5c* expression at a PELOD-2 score on postoperative day 2 of 6, with levels also lower at a score of 7 (Figure 2A). Additionally, we demonstrated a strong negative correlation between *myo5*c expressions upon ICU admission and PELOD-2 scores on postoperative day 2 (*r* = −0.6393, *p* < 0.0001) (Figure 2B). *Myo5c* expression also negatively correlated with the ICU length of stay (*r* = −0.3030, *p* = 0.0325) (Figure 2C). The receiver operating characteristic (ROC) curve showed an acceptable discriminatory ability of *myo5c* expression upon ICU admission in relation to PELOD-2 score on postoperative day 2 (AUROC = 0.6754, 95% CI 0.56–0.79, *p* = 0.0025) (Supplementary Figure S2).

We further assessed whether *myo5c* expression declined after surgery by comparing baseline and ICU admission levels within each group. No significant within-group differences were found in either the complication or non-complication group (Supplementary Figure S3). Additionally, logistic regression models were constructed to assess whether the association between *myo5c* expression and postoperative complications was confounded by other clinical variables. In all models, only STAT category ≥3 emerged as a significant independent predictor of complications, while *myo5c* expression was not statistically significant (Supplementary Tables S1, S2).

In addition, we performed subgroup analyses to determine whether *myo5c* expression was influenced by lesion type or surgical complexity. Patients were stratified by cyanotic vs. acyanotic CHD, as well as by STAT mortality category. No significant differences in *myo5c* expression were observed between cyanotic and acyanotic groups at either baseline or ICU admission (Supplementary Figure S4). Similarly, no consistent trend was identified across the full range of STAT categories (1–5) (Supplementary Figure S5). In contrast, when patients were grouped by STAT mortality category <3 vs. ≥3, *myo5c* expression at ICU admission showed a downward trend in the STAT ≥3 group, though this did not reach statistical significance (Supplementary Figure S6).

However, given the limited sample size in higher STAT categories, these results are considered exploratory and require validation in larger, stratified cohort.

### Myo5C gene expression was negatively correlated with IL-6 release, neutrophil and platelet activations

To further elucidate the association between *myo5c* expression and the inflammatory response, we conducted an analysis examining the correlation of *myo5c* expression levels with various parameters, including cytokine release, neutrophil and platelet activation, as well as neutrophil and platelet counts.

**Figure 3:**
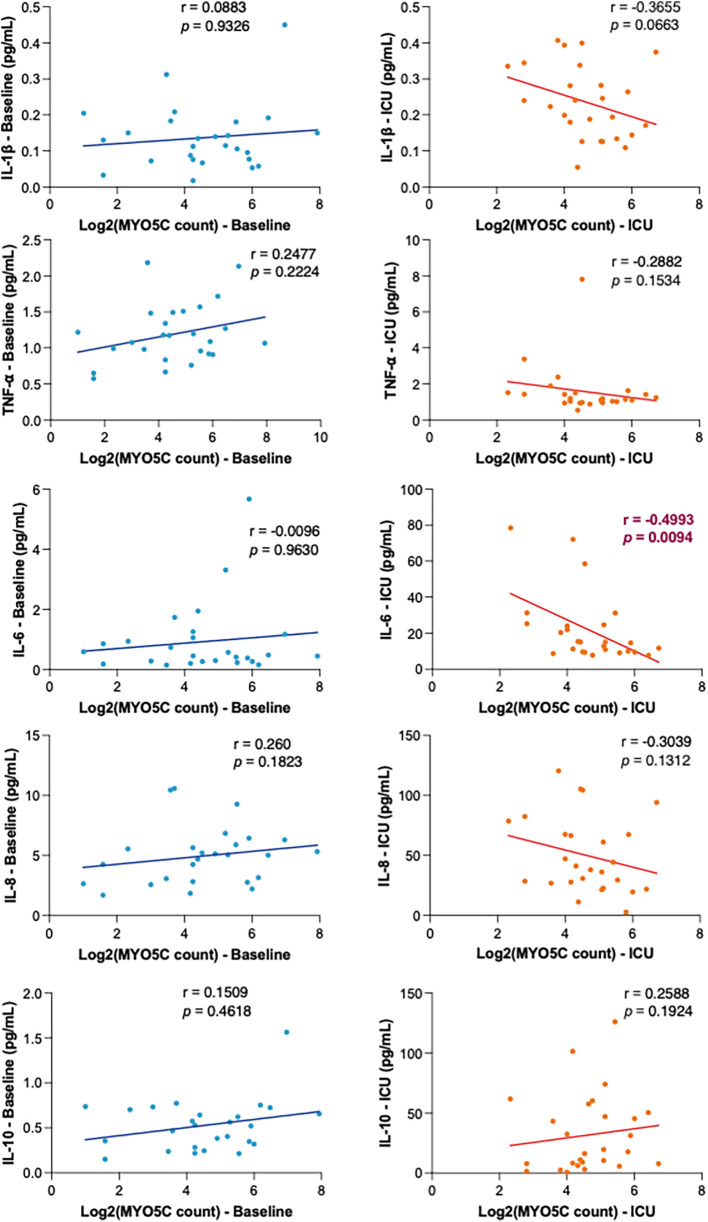
Correlation analyses of *myo5c* gene expression in inflammatory response. Correlation of *myo5c* gene expression and cytokine levels (IL-1β, TNF-α, IL-6, IL-8, and IL-10 top to bottom) at baseline (blue) and upon ICU admission (red) was determined using linear regression analysis. Spearman correlation coefficients (r) and corresponding *p*-values are shown for each marker.

**Figure 4:**
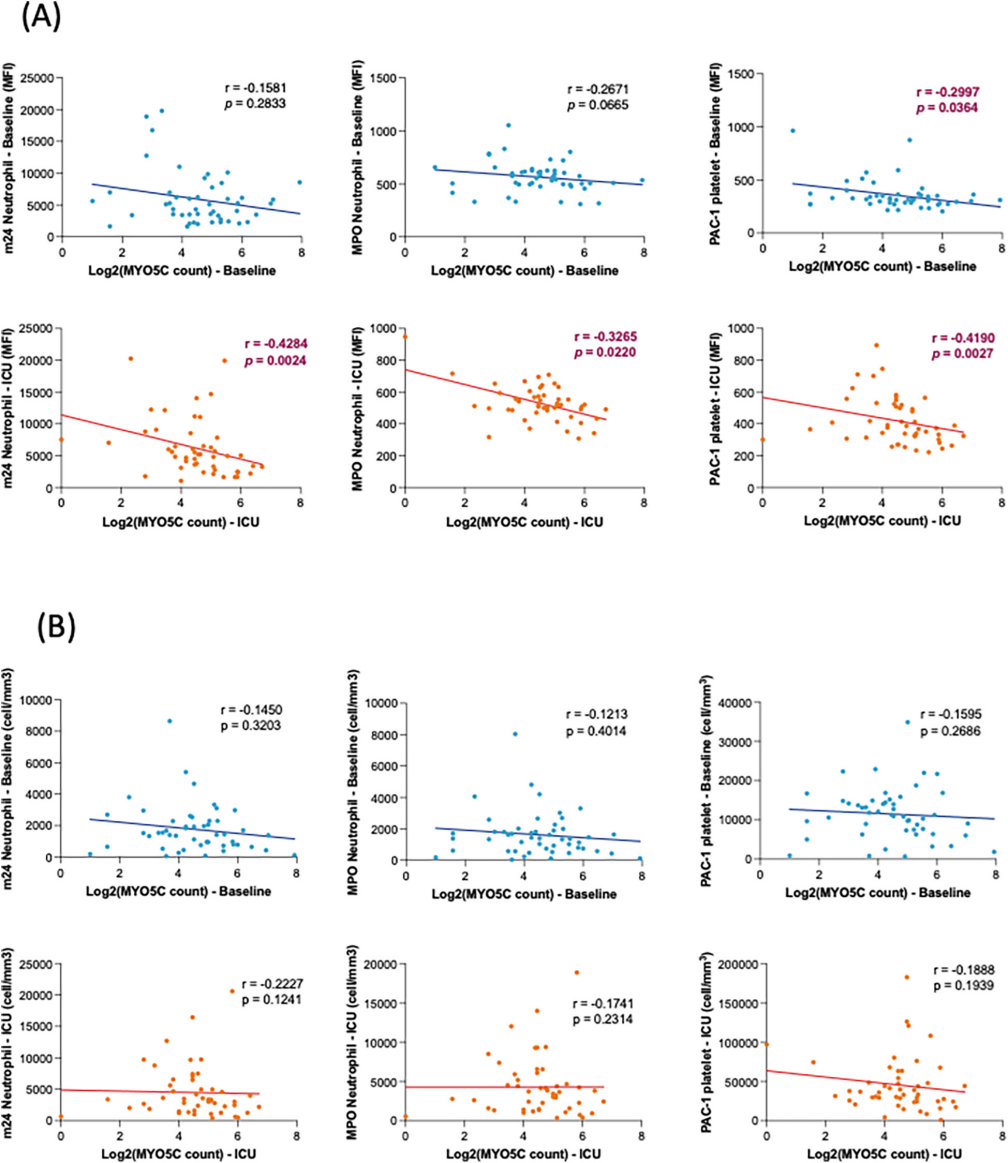
Correlation analyses of *myo5c* gene expression with neutrophil/platelet activation. **(A)** Correlation between *myo5c* gene expression and neutrophil and platelet activation at baseline (blue) and upon ICU admission (red). Neutrophil activation was assessed by m24 and MPO staining; platelet activation was measured by PAC-1 staining. Activation levels are represented as mean fluorescence intensity (MFI). **(B)** Correlation of *myo5c* gene expression and the number of activation marker-positive neutrophils (m24, MPO) and platelets (PAC-1) activation at baseline (blue) and upon ICU admission (red). Cell counts (cells/mm³) represent the number of marker-positive cells, measured by flow cytometry. **(A,B)** Spearman correlation analysis was used, and correlation coefficients (*r*) and corresponding *p*-values are shown.

Our analysis revealed a statistically significant negative correlation between *myo5c* expression and organ injury marker, IL-6 levels upon ICU admission (*r* = −0.4993, *p* = 0.0094) (Figure 3). Additionally, elevated *myo5c* expression was significantly associated with less activation of both neutrophils (at T2, m24 vs. *myo5c*, *r* = −0.4284, *p* + 0.0024) and platelets (at T2, PAC1 vs. *myo5c*, *r* = −0.4190, *p* = 0.0027) (Figure 4A). However, neutrophil and platelet counts did not exhibit a statistically significant correlation (Figure 4B). These findings supported the hypothesis that *myo5c* potentially play a substantial role in the regulation of systemic inflammatory response following CPB.

### Myo5C deficiency enhanced neutrophil extracellular trap (NET) formation

**Figure 5:**
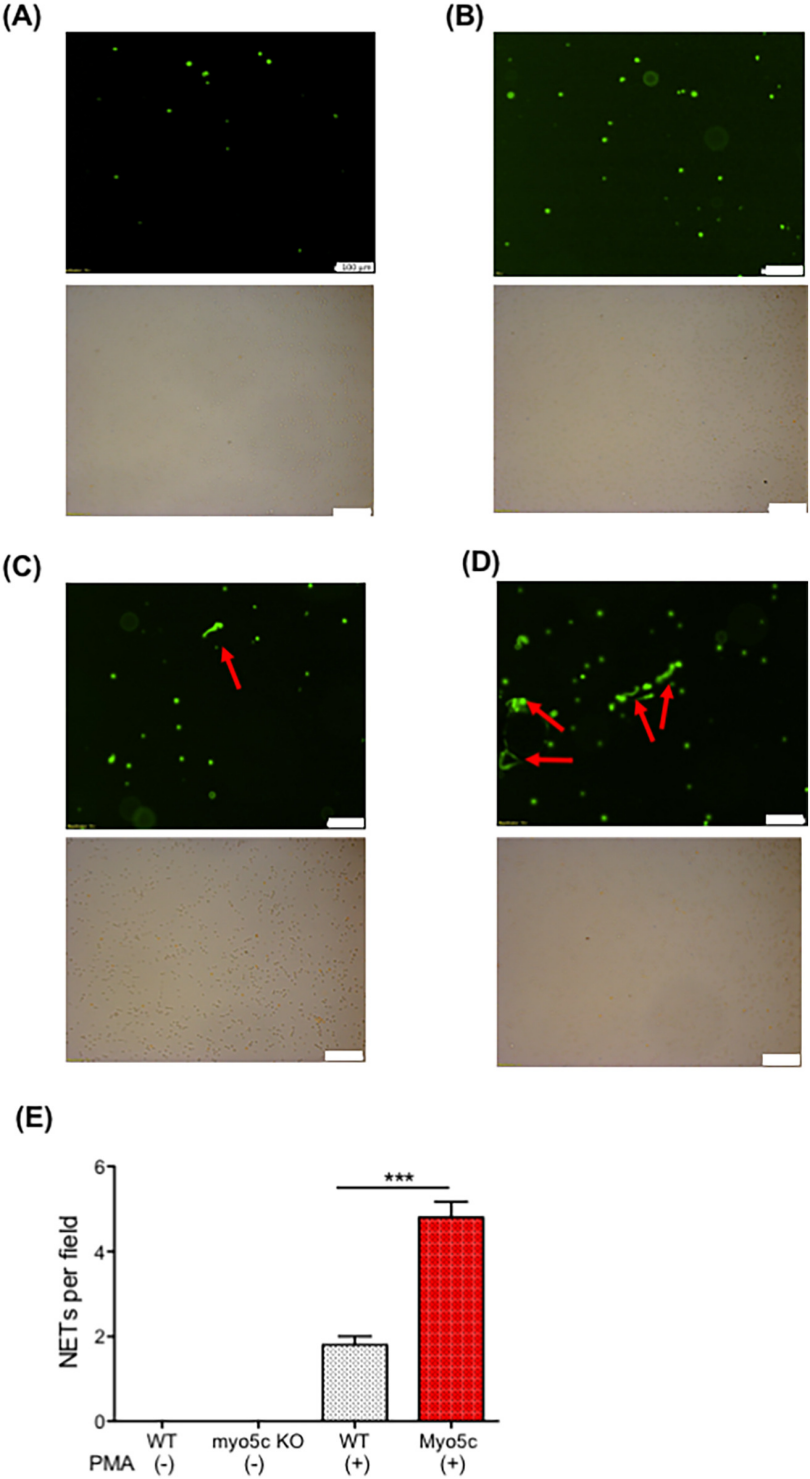
The role of *myo5c* in NETs formation. NETs formation was examined using neutrophil-differentiated HL-60 cells. Neutrophil-differentiated HL-60 cells were subjected to NETs induction by incubating with PMA for 4 h. **(A)** HL60 WT cells. **(B)** HL60 *myo5c* KO, **(C)** HL60 + PMA, **(D)** HL60 *myo5c* KO + PMA. Top image shows Sytox green image. The bottom shows bright-field image. The white bar denotes 100 µm. **(E)** The number of NETs formation per field in the five independent experiments was counted and shown. Data are representative of the two independent experiments. One-way ANOVA was performed. *** *p* < 0.001.

The observed downregulation of the neutrophil *myo5c* gene in patients with elevated PELOD-2 scores suggested its potential involvement in postoperative complications. To determine the functional role of *myo5c*, we performed its deletion in HL60 cells using CRISPR/Cas9. Our investigation into NET formation in HL60 cells with *myo5c* knockout phenotypes revealed enhanced NET formation in the absence of *myo5c*protein following PMA stimulation (Figure 5), suggesting that *myo5c* would be a functionally important molecule. This experimental evidence supported our initial hypothesis, strengthening the association between *myo5c*, systemic inflammation, and NET formation.

## Discussion

Our study identifies a potential role for *myo5c* in modulating neutrophil activation and inflammation in pediatric patients undergoing cardiac surgery with CPB. Reduced *myo5c* expression in circulating neutrophils was associated with greater postoperative organ dysfunction, as reflected by higher PELOD-2 scores, increased surgical complexity (STAT mortality category ≥3), and prolonged ICU and hospital stays. Although *myo5c* expression was not independently associated with the presence of complications, its strong inverse correlation with PELOD-2 scores suggests a closer relationship with the severity of physiological stress and evolving organ injury.

Functionally, *myo5c*-deficient HL60 cells exhibited enhanced NET formation upon PMA stimulation, supporting a role of Myosin Vc protein in regulating neutrophil activation. Transcriptomic downregulation of *myo5c* also correlated with elevated IL-6 levels, and markers of neutrophil and platelet activation, further linking reduced *myo5c* expression to a proinflammatory and potentially prothrombotic phenotype.

Class V myosin, key member of the myosin superfamily, serve as actin-based molecular motors essential for cellular motility and organelle transport (17–19). These myosin V proteins are ubiquitous and fundamental in establishing the molecular mechanisms of actin-mediated cellular activities (20). In vertebrates, the class V myosin family comprises three genes: *myo5a*, *myo5b*, and *myo5c* (21–23). While the roles of Myosin Va and Vb proteins have been extensively studied, Myosin Vc remains less investigated. This novel variant, prevalent in secretory and glandular tissues, is implicated in diverse cellular functions such as secretory granule trafficking (24), transferrin trafficking (22), melanosome biogenesis and secretion (25), and von Willebrand factor (vWF) externalization (26). Despite its presence in various immune cells, including neutrophils (27, 28), the specific roles of Myosin Vc in immune responses are not well-defined.

Our findings suggest that *myo5c* downregulation may be associated with postoperative organ dysfunction via enhanced neutrophil activation and NET formation, which in turn may promote vascular microthrombosis, impair tissue perfusion, and contribute to subsequent organ injury (29, 30). While direct evidence of Myosin Vc's role in NET formation is lacking, its established functions in actin cytoskeletal rearrangement may be important for chromatin decondensation and release of nuclear DNA during NET formation (31, 32). In our study, *myo5c*-knockout cells produced more NETs, suggesting a previously unrecognized role for Myosin Vc in neutrophil effector functions.

Following cardiac surgery with CPB, extensive tissue injury and cellular damage leads to the release of damage-associated molecular patterns (DAMPs), such as histones and high mobility group box 1 (HMGB1), which activate neutrophils (33) and trigger their degranulation and NET release (34, 35). Our previous work demonstrated elevated circulating DAMPs in patients with postoperative organ dysfunction and showed that DAMPs enhance NET formation in a mouse model (3). These findings underscore the relevance of NET-driven mechanisms in postoperative organ dysfunction and raise the possibility that Myosin Vc may modulate this response.

Taken together, these cellular processes may help explain the observed association between lower *myo5c* expression and higher PELOD-2 scores, linking transcriptomic alterations with postoperative organ dysfunction severity. Although our study did not include direct imaging of actin remodeling or vesicle transport, future mechanistic studies will be essential to define how Myosin Vc regulates neutrophil activation and NET formation. To avoid overinterpretation, we emphasize that this represents a hypothesis-generating observation based on indirect mechanistic parallels from correlative data and *in vitro* observations, and additional research is needed to validate the proposed pathway.

This study has several limitations. It was exploratory in nature, with no formal sample size calculation. The relatively small sample size, modest number of complication cases, and single-center design may reduce statistical power and limit generalizability. The ROC AUC for *myo5c* expression indicates moderate discriminatory ability and should be interpreted with caution. Additionally, heterogeneity in CHD diagnoses and surgical complexity may influence gene expression. Nevertheless, *myo5c* downregulation may have clinical value as part of a multi-marker panel for early risk stratification. Future studies with larger, more homogeneous cohorts are needed to validate the clinical utility of *myo5c* and elucidate its mechanistic role in inflammation.

In conclusion, this study highlights the association between *myo5c* gene expression, neutrophil activation, and postoperative organ dysfunction severity in pediatric cardiac surgery with CPB. Our findings support the potential role of *myo5c* in modulating NET formation and systemic inflammation. While *myo5c* was not identified as an independent predictor of clinical outcomes, its consistent association with markers of illness severity suggests it may serve as a valuable biomarker, particularly as part of a multi-marker panel, for early risk stratification. Further research is warranted to elucidate the regulatory mechanisms underlying *myo5c* expression and to explore the molecular regulation and functional relevance of Myosin Vc protein in neutrophil-mediated inflammation.

## Data Availability

The original contributions presented in the study are publicly available. This data can be found here: https://www.ncbi.nlm.nih.gov/geo/query/acc.cgi?acc=GSE297377.

## Supplementary material

Supplementary Figure S1 Neutrophils and platelets gating strategy. **(A)** Neutrophils gated by CD15(+) population. Activation antibody m24 was gated among the neutrophils. **(B)** Platelets gated by CD41 (+) population. Activation antibody PAC-1 was gated among the platelets.

Supplementary Figure S2 Receiver Operating Characteristic (ROC) curve analysis comparing discrimination performance of *myo5c* expression upon ICU admission and PELOD-2 score on postoperative day 2.

Supplementary Figure S3 *Myo5c* expression in neutrophils stratified by complication status. Log_2_-transformed *myo5c* counts at baseline (blue) and upon ICU admission (red) in patients with **(A)** and without **(B)** postoperative complications. *ns* = not significant.

Supplementary Figure S4 *Myo5c* expression in neutrophils stratified by cyanosis status. Log_2_-transformed *myo5c* counts at baseline (blue) and upon ICU admission (red) in acyanotic vs. cyanotic patients. *ns* = not significant.

Supplementary Figure S5 *Myo5c* expression across full STAT mortality categories (1–5). Box plots showing expression at baseline (blue) and upon ICU admission (red). *ns* = not significant.

Supplementary Figure S6 *Myo5c* expression in STAT category <3 vs. ≥3. Box plots showing expression at baseline (blue) and upon ICU admission (red). *ns* = not significant.

Supplementary Table S1 Logic regression analysis of baseline *myo5c* expression and clinical variables.

Supplementary Table S2 Logic regression analysis of *myo5c* expression at ICU admission and clinical variables.
