# Supplementary material for: Neutrophil Myo5c gene downregulation is associated with postoperative organ dysfunction following pediatric cardiac surgery with cardiopulmonary bypass
Source: Front Cardiovasc Med. 2025 May 27;12:1380606. doi: 10.3389/fcvm.2025.1380606 (PMC12148857; doi:10.3389/fcvm.2025.1380606)
Supplement: Supplementary Table S2 — Logic regression analysis of myo5c expression at ICU admission and clinical variables. [file Datasheet1.pdf]

Supplementary Material

Neutrophil *Myo5c* Gene Downregulation is Associated with Postoperative Organ Dysfunction Following Pediatric Cardiac Surgery with Cardiopulmonary Bypass.

Running title: Neutrophil *myo5c* gene & pediatric cardiac surgery

Wiriya Maisat, Sumiti Sandhu, Samuel Kim, Hanna Van Pelt, Sek Won Kong, Juan Ibla
Koichi Yuki

Supplementary methods

Definition of postoperative complication

Postoperative complication was defined as organ dysfunction/injury and/or thrombosis. Due to the lack of a standardized definition for organ dysfunction post-congenital cardiac surgery, we used previously published by others (2, 3, 4). Organ dysfunctions included 1) cardiovascular dysfunction (low cardiac output syndrome reliant on a vasoactive drug to maintain blood pressure, or two of the followings; metabolic acidosis, elevated arterial lactate, oliguria, or prolonged capillary refill), 2) respiratory dysfunction (arterial oxygen tension/fraction of inspired oxygen (P_aO_2/F_iO_2) <300, arterial carbon dioxide tension (P_aCO_2) >65 torr or 20 mmHg over baseline P_aCO_2, need for >50% F_iO_2 to maintain oxygen saturation ≥92%, or need for non-elective mechanical ventilation, prolonged mechanical ventilation ≥5 d, 3) renal dysfunction (the presence of creatinine of >114 μmol/L, urine output of <1 mL/kg/hr despite diuretic administration, or the requirement for ultrafiltration or hemodialysis 4) coagulopathy or bleeding complication requiring chest exploration for bleeding or removal of clots, intracranial hemorrhage, prothrombin or partial thromboplastin time was three times normal, or >30 mL/kg of blood products were infused during a 24-hr period, 5) central nervous system dysfunction (development of a new intracranial infarct or hemorrhage, evidence of hypoxic-ischemic injury by clinical examination or computed tomography of the head, or brain death), 6) hepatic dysfunction (bilirubin concentration of >2 mg/dL and/or increase of hepatic cellular enzymes two or more times normal). Thrombosis was defined as the presence of any vascular thrombosis detected using ultrasound diagnostic imaging.

Neutrophil and platelet activation

Neutrophil and platelet counts were determined from 10 μL of whole blood collected. The blood was first incubated with an Fc receptor-blocking solution and then stained with fluorescent-conjugated antibodies against CD15 (neutrophil marker), CD18 (clone m24), and myeloperoxidase (MPO) for neutrophils (**Suppl. Fig. 1A**), and CD41 (platelet marker) and PAC-1 for platelets (Biolegend Inc., San Diego, CA, USA) (**Suppl. Fig. 1B**). After a 30-minute incubation, the stained samples were treated with 1% FACS™ Lysing Solution (BD Bioscience, San Jose, CA, USA). The cells were resuspended in phosphate-buffered saline (PBS) and analyzed using a BD Accuri C6 flow cytometer (BD Biosciences, Franklin Lakes, NJ, USA). Because m24 is an activation-sensitive antibody against CD18 (5), activated neutrophils were identified based on the expression of CD15 and m24 (CD18) or MPO. Because PAC-1 is an activation-sensitive antibody against platelet integrin αIIbβ3 (6), activated platelets were identified through CD41 and PAC-1 expression. A total of 100 μL was processed for each analysis.

Neutrophil isolation and mRNA extraction

Neutrophils were isolated from whole blood samples using Polymorphprep™ for density gradient separation, per the manufacturer's instruction (AXIS-SHIELD PoC AS, Oslo, Norway). In brief, fresh citrated whole blood was layered atop an equal volume of Polymorphprep™ (Proteogenix, Miami, FL, USA) and then centrifuged at 500 x *g* for 30 minutes. The neutrophil layer was collected for RNA extraction using Trizol reagent (Life Technologies, Carlsbad, CA, USA), following the manufacturer's protocol.

RNA sequencing analysis of neutrophil mRNA

RNA sequencing was performed using Illumina TruSeq Stranded Total RNA sample preparation, followed by next-generation sequencing (NGS) using Illumina NovaSeq to generate 50 million 150bp paired-end reads per sample. Library preparation and NGS were performed at the Harvard Institute of Medicine. This sequencing depth provides abundant sensitivity for all expressed transcripts, both protein-coding and regulatory (microRNA and long non-coding RNA), including exon-exon junction reads to detect differential usage of splicing variants. The analysis proceeded using a standard bioinformatics pipeline developed for profiling transcript-level abundance in bulk RNA-seq. The quality of the raw reads was evaluated using FastQC (v0.11.9). Any detected Nextera transposase adapter contaminations within the reads were trimmed using TrimGalore (v0.6.10). The reads were then aligned to the human reference genome GRCh38 using the 2-PASS STAR algorithm (v2.7.10b). HTseq-count (v2.0.2) was employed to generate raw counts for each transcript. Inter-individual differences were significantly more pronounced than the differences between pre and post-surgery states. A differential expression analysis was conducted on the log2-normalized expression data using a mixed-effect model with the lmerSeq R package to account for this subject-level variability. This model compared post-surgery states to pre-surgery ones. The Bonferroni correction was applied to adjust p-values for multiple comparisons. Genes that exhibited a false-discovery rate less than 0.05 and an absolute fold change greater than 1.5 were considered significant. Gene ontology (GO) Enrichment analysis was performed to demonstrate the function of significant differentially expressed genes (DEGs) in Biological Process, and Kyoto Encyclopedia of Genes and Genomes (KEGG) pathway were examined using the Database for Annotation, Visualization and Integrated Discovery (DAVID) online analysis tool (7), with adjusted *p*-values calculated using the Benjamini-Hochberg correction to control for multiple comparisons.

Myo5c knockout HL-60 cells

CRISPR/Cas9 editing to delete *myo5c* expression was done in HL-60 cells. HL-60 cells were cultured at 37°C in RPMI1640 supplemented with 10% FBS, 1% penicillin/streptomycin. Confluency was maintained between 3x 10^5 -1.5 x 10^6 cells/mL. Electroporation was performed one day after passaging when cells were in log phase of growth using the Lonza 4D Nucleofector with 20 µL Nucleocuvette strips as we did before (8, 9). Ribonucleoprotein (RNP) complex was made by combining 100 pmol Cas9 (IDT; Newark, NJ) and 100 pmol modified sgRNA (Synthego; Redwood City, CA) targeting *myo5c* using either sg1 (TCCTGTTGGTCAGTACAACA) or sg2 (TTGGTCAGTACAACAGGGTC) and incubating at room temperature for 15 minutes. As a control, sgRNA targeting AAVS1 was used (GGGGCCACTAGGGACAGGAT). 2x10^5-4x10^5 HL-60 cells were resuspended in 20µL SF cell line solution (Lonza; Basel, Switzerland) and mixed with RNP. They were subjected to nucleofection with program EN-138 as per manufacturer recommendation, Cells were returned to RPMI1640 media and editing efficiency was measured 48 hours after electroporation by genomic polymerase chain reaction (PCR) followed by Sanger sequencing.

HL-60 neutrophil differentiation and in vitro NETs assay

HL-60 cells were subjected to neutrophil differentiation in 1.25% DMSO and 1 µM all-trans retinoic acid (ATRA, Sigma-Aldrich; St. Louis, MO) for 4 days. Then, they were stimulated with phorbol myristate acetate (PMA) at 37°C for 4 hours. To quantify NETs, the cells at the termination of NET induction were washed once and then Sytox green (0.1 µM) was added. The NETs were imaged with a fluorescence microscope, Zeiss Axiovert (Carl Zeiss, Thornwood, NY).

PELOD-2 score calculation

Organ dysfunction and variables[a]	Points by severity levels						
	0	1	2	3	4	5	6
Neurologic[b]							
Glasgow Coma Score	≥11	5-10			3-4		
Pupillary reaction	Both reactive					Both fixed	
Cardiovascular[c]							
Lactatemia (mmol/L)	<5.0	5.0-10.9			≥11.0		
MAP (mmHg)							
0 to <1 mo	≥46		31-45	17-30			≤16
1-11 mo	≥55		39-54	25-38			≤24
12-23 mo	≥60		44-59	31-43			≤30
24-59 mo	≥62		46-61	32-44			≤31
60-143 mo	≥65		49-64	36-48			≤35
≥144 mo	≥67		52-66	38-51			≤37
Renal							
Creatinine (μmol/L)							
0 to <1 mo	≤69		≥70				
1-11 mo	≤22		≥23				
12-23 mo	≤34		≥35				
24-59 mo	≤50		≥51				
60-143 mo	≤58		≥59				
≥144 mo	≤92		≥93				
Respiratory[d]							
PaO_2 (mmHg)/FiO_2	≥61		≤60				
$PaCO_2$ (mmHg)	≤58	59-94		≥95			
Invasive ventilator	No			Yes			
Hematologic							
WBC count (x 10^9/L)	>2		≤2				
Platelet (x 10^9/L)	≥142	77-141	≤76				

[a] All variables must be collected, but measurements can be done only if justified by the patient's clinical status. If a variable is not measured, it should be considered normal. If a variable is measured more than once in 24 hr, the worst value is used in calculating the score. Fio2: fraction of inspired oxygen.

[b] Neurologic dysfunction: Glasgow Coma Score: use the lowest value. If the patient is sedated, record the estimated Glasgow Coma Score before sedation. Assess only patients with known or suspected acute central nervous system disease. Pupillary reactions: nonreactive pupils must be > 3 mm. Do not assess after iatrogenic pupillary dilatation.

[c] Cardiovascular dysfunction: Heart rate and mean arterial pressure: do not assess during crying or iatrogenic agitation.

[d] Respiratory dysfunction: Pao2: use arterial measurement only. Pao2/Fio2 ratio is considered normal in children with cyanotic heart disease. Paco2 can be measured from arterial, capillary, or venous samples. Invasive ventilation: the use of mask ventilation is not considered invasive ventilation.

Supplementary Table 1. Logic regression analysis of baseline *myo5c* expression and clinical variables.

Variable	Adjusted OR (95%Ci)	p value
STAT mortality category ≥3	7.44 (1.65-33.54)	0.009
Myo5c expression at baseline	0.68 (0.40-1.14)	0.14

Supplementary Table 2 Logic regression analysis of *myo5c* expression at ICU admission and clinical variables.

Variable	Adjusted OR (95%Ci)	p value
STAT mortality category ≥3	5.31 (1.42-19.90)	0.013
Myo5c expression at ICU	0.72 (0.43-1.21)	0.22

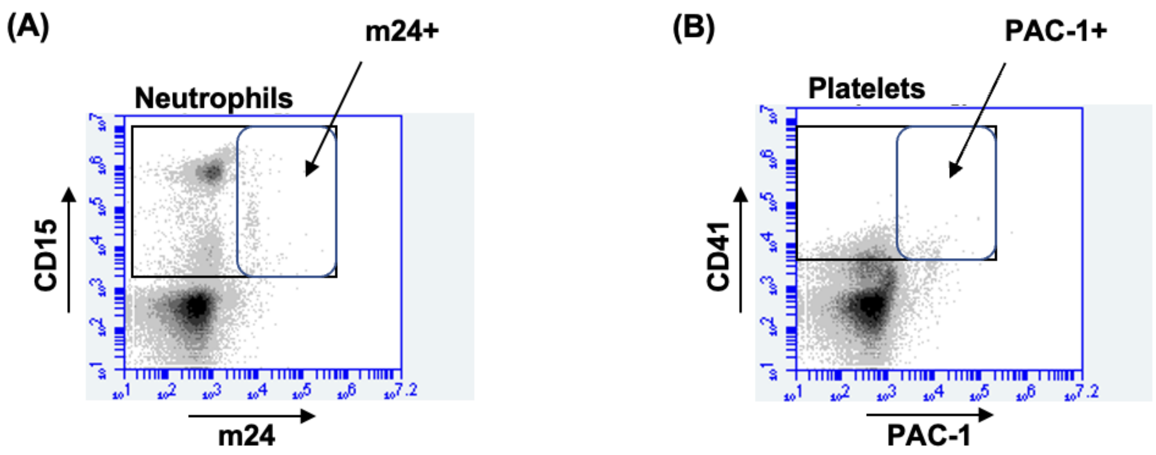

Supplementary Figure 1. **Neutrophils and platelets gating strategy**

A. Neutrophils gated by CD15(+) population. Activation antibody m24 was gated among the neutrophils. B. Platelets gated by CD41 (+) population. Activation antibody PAC-1 was gated among the platelets.

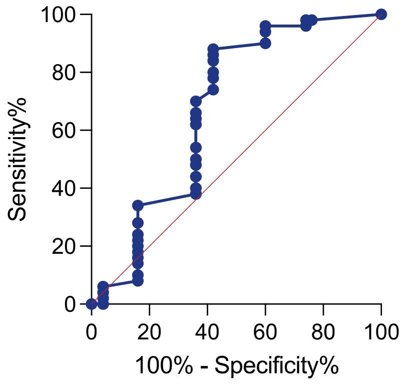

Supplementary Figure 2. Receiver Operating Characteristic (ROC) curve analysis comparing discrimination performance of *myo5c* expression upon ICU admission and PELOD-2 score on postoperative day 2.

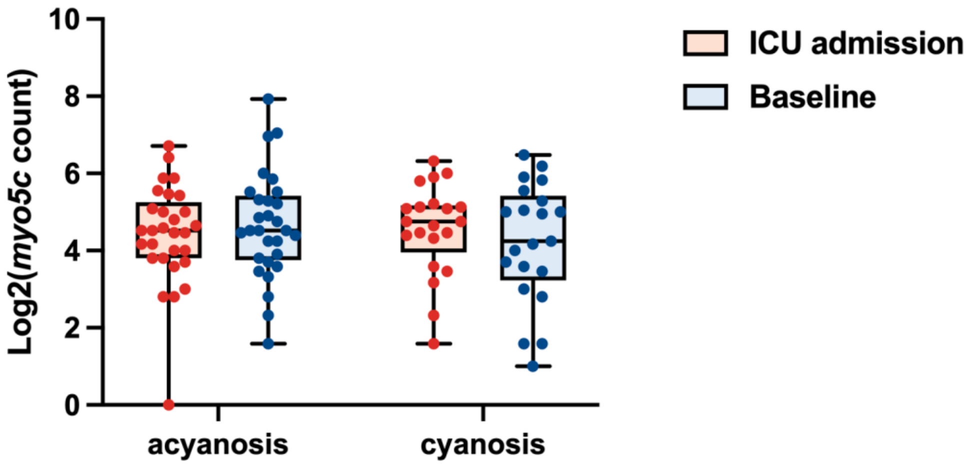

Supplementary Figure 3.

Myo5c expression in neutrophils stratified by cyanosis status. Log2-transformed *myo5c* counts at baseline (blue) and upon ICU admission (red) in acyanotic vs. cyanotic patients.

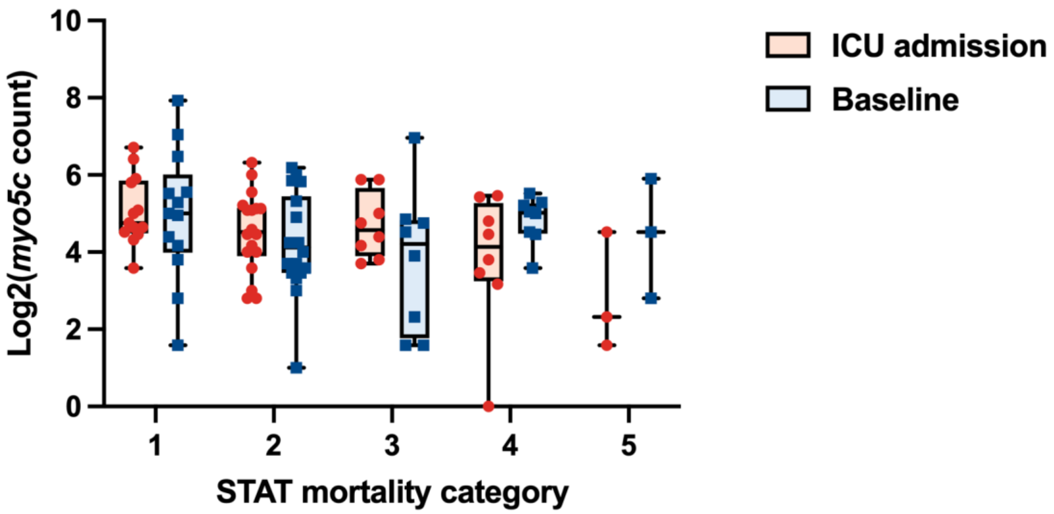

Supplementary Figure 4.

Myo5c expression across full STAT mortality categories (1-5). Box plots showing expression at baseline (blue) and upon ICU admission (red).

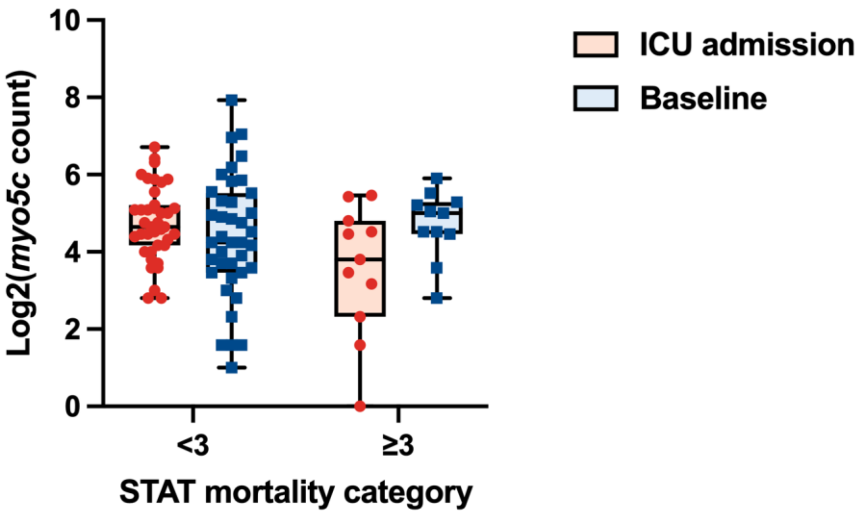

Supplementary Figure 5.

Myo5c expression in STAT category <3 vs. ≥3. Box plots showing expression at baseline (blue) and upon ICU admission (red).

References

1. Leteurtre S, Duhamel A, Salleron J, Grandbastien B, Lacroix J, Leclerc F. PELOD-2: an update of the PEdiatric logistic organ dysfunction score. Crit Care Med. 2013;41(7):1761-73.
2. Montgomery VL, Strotman JM, Ross MP. Impact of multiple organ system dysfunction and nosocomial infections on survival of children treated with extracorporeal membrane oxygenation after heart surgery. Crit Care Med. 2000;28(2):526-31.
3. Seghaye MC, Engelhardt W, Grabitz RG, Faymonville ME, Hörnchen H, Messmer BJ, et al. Multiple system organ failure after open heart surgery in infants and children. Thorac Cardiovasc Surg. 1993;41(1):49-53.
4. Bembea MM, Agus M, Akcan-Arikan A, Alexander P, Basu R, Bennett TD, et al. Pediatric Organ Dysfunction Information Update Mandate (PODIUM) Contemporary Organ Dysfunction Criteria: Executive Summary. Pediatrics. 2022;149(1 Suppl 1):S1-s12.
5. Sen M, Yuki K, Springer TA. An internal ligand-bound, metastable state of a leukocyte integrin, alphaXbeta2. J Cell Biol. 2013;203(4):629-42.
6. Yuki K, Bu W, Shimaoka M, Eckenhoff R. Volatile anesthetics, not intravenous anesthetic propofol bind to and attenuate the activation of platelet receptor integrin alphaIIbbeta3. PLoS One. 2013;8(4):e60415.
7. Sherman BT, Hao M, Qiu J, Jiao X, Baseler MW, Lane HC, et al. DAVID: a web server for functional enrichment analysis and functional annotation of gene lists (2021 update). Nucleic Acids Res. 2022;50(W1):W216-W21.
8. Hou L, Voit RA, Shibamura-Fujiogi M, Koutsogiannaki S, Li Y, Chen Y, et al. CD11c regulates neutrophil maturation. Blood Adv. 2023;7(7):1312-25.
9. Koutsogiannaki S, Hou L, Okuno T, Shibamura-Fujiogi M, Luo HR, Yuki K. alphaDbeta2 as a novel target of experimental polymicrobial sepsis. Front Immunol. 2022;13:1059996.
